# Vitamin D level could affect the recovery rate in traumatic brain injury: a retrospective study

**DOI:** 10.1186/cc13661

**Published:** 2014-03-17

**Authors:** FI Socci, S Di Valvasone, A Cecchi, M Bonizzoli, A Terreni, A Peris

**Affiliations:** 1 AOU Careggi, Florence, Italy

## Introduction

Recent studies have shown that 1,25-dihydroxyvitamin D3 (vitamin D) deficiency may affect negatively the clinical course of traumatic brain injury (TBI) [1]. This problem becomes important with respect to the older patient considering a 50% prevalence of vitamin D deficiency [2]. Data from the Third National Health and Nutrition Examination Survey [3] document more than 60% of Caucasians affected by D deficiency [4] so that all patients with TBI of any age are theoretically at risk of unfavorable outcome [2]. The objective of this preliminary study was to determine whether low levels of vitamin D at admission to the ICU (<24 hours) could negatively affect neurological recovery of patients with TBI.

## Methods

We retrospectively analyzed the data of 46 patients affected by TBI (65% severe, 9.5% moderate, 28.5% moderate) both isolated or associated with other extracranial lesions. The sampling of vitamin D was carried out within 24 hours from ICU admission. We had registered GCS at the moment of presentation (GCS in) and at discharge (GCS out) and their difference (GCS diff) compared with levels of vitamin D. Patients that died in the ICU were assigned a GCS out = 0. See Table 1.

**Table 1:** 

			TBI		
				
Descriptive statistics			Isolated	Associated	*P *value
Dead	8.70%	Percentage	22	78	
Male	87%	GCS ir	4 ± 3	8 ± 5	0.043
Age	50 ± 21	GCS out	10 ± 4	10 ± 5	0.933
BMI	26.36 ± 4.14	GCS diff	6 ± 4	3 ± 6	0.151
ISS	29 ± 15	Vitamin D (ng/ml)	17 ± 8.5	17 ± 9.8	0.597
Vitamin D deficiency (<30 ng/ml)	73.90%	Length of stay ICU (days)	12 ± 7	8 ± 9	0.261

## Results

Our data, according to other studies [5], confirm the presence of a deficiency of vitamin D (Table 1); however, they do not demonstrate a statistical significance correlation at the univariate regression *(R *= 0.04; *P *= 0.786) between vitamin D level and outcome from the ICU. There was no correlation stratifying patients for age, for TBI class, for Injury Severity Score and for BMI.

## Conclusion

Vitamin D deficiency is really prevalent in our TBI cases but does not seem to affect neurological recovery at ICU discharge; however, these preliminary results should be exposed to several criticisms and need to be confirmed with prospective studies.
